# Microbiome–volatile metabolome analysis reveals aroma regulation driven by microbial niche competition in Jinggang honey pomelo wine

**DOI:** 10.3389/fmicb.2025.1725554

**Published:** 2026-01-02

**Authors:** Yang Wu, Weiwei Li, Jianan Shi, Zhihao Zhang, Cheng Jing, Jianqi Sheng, Zexia Li, Xiaowen Shi, Dingkun Liu, Li He, Huimin Sun

**Affiliations:** 1 School of Life Sciences, Jinggangshan University, Ji’an, China; 2 Key Laboratory of Jiangxi Province for Biological Invasion and Biosecurity, School of Life Sciences, Jinggangshan University, Ji’an, China; 3 Key Laboratory of Jiangxi Province for Functional Biology and Pollution Control in Red Soil Regions, School of Life Sciences, Jinggangshan University, Ji’an, China; 4 Agricultural and Rural Industry Development Service Center, Ji’an, China; 5 Technology Innovation and Development Center, Ji’an, China

**Keywords:** Jinggang honey pomelo wine, semi-artificial inoculation fermentation, microbial niche competition, volatile compounds, correlation analysis

## Abstract

**Introduction:**

Microbial succession in fruit wine has been reported, but the ecological mechanisms linking niche competition to aroma formation remain poorly understood. To test the hypothesis that niche competition between microbial communities significantly influences aroma formation in pomelo wine, the flesh of Jinggang honey pomelo was subjected to semi-inoculation fermentation to produce Jinggang honey pomelo wine.

**Methods:**

High-throughput amplicon sequencing technology was used to investigate the evolving microbial community during the fermentation process of pomelo wine. The changes in volatile compounds were measured using headspace solid phase microextraction (HS-SPME) coupled with gas chromatography-mass spectrometry (GC-MS).

**Results:**

The dominant taxa in the wine were *Weissella*, *Pediococcus*, *Lactiplantibacillus*, *Saccharomyces*, *Komagataella*, *Wickerhamomyces*, and *Aspergillus*. The microbiota shifts were associated with dynamic changes in physicochemical properties, and they altered the pH, alcohol content, total soluble solids, and overall acidity. Principal component analysis (PCA), orthogonal partial least squares-discriminant analysis (OPLS-DA), and relative odor activity value analysis identified 17 key volatiles. A correlation network analysis revealed that *Lactiplantibacillus* and *Aspergillus* were strongly associated with various flavor molecules.

**Disscussion:**

The present findings suggested that inter-kingdom niche competition between fungi and bacteria plays a pivotal role in shaping the aroma profile of pomelo wine, offering new insights for targeted aroma regulation.

## 1 Introduction

The process of fermenting wine involves various microorganisms, such as yeast, bacteria, and filamentous fungi (Wei et al., 2022). The wine microbial community is mainly derived from the pulp. Owing to the metabolism of nutrients, such as sugar, and the production of alcohol, organic compounds, and antibacterial compounds, the oxidation and fermentation species communities that constitute the wine microbial community continuously evolve (Bisson and Walker, 2015; Ding et al., 2021; Englezos et al., 2022; Renouf et al., 2005). During fermentation, these microbes generate certain active metabolic compounds that result in the buildup of secondary aroma compounds, ultimately impacting the aromatic complexity and sensory qualities of wine (Di Cagno et al., 2017). Aroma formation in fermented beverages is increasingly understood as an emergent property of microbial cross-kingdom interactions that occupy and partition ecological niches along the fermentation continuum.

Microbial genomics is the core technology for analyzing the microbial community of fruit wine fermentation. Microbial genomics can comprehensively identify bacteria and fungi in samples without cultivation, accurately track the dynamic succession of microbial communities, and provide key insights into the microbiological driving mechanisms of fermentation flavor (Gao et al., 2023; Liu et al., 2020; Pinto et al., 2021; Pinto et al., 2015; Portillo et al., 2016). Volatile metabolomics has become a cornerstone for deciphering the biochemical basis of wine aroma, enabling real-time monitoring and targeted modulation of fermentation outcomes (Kinzurik et al., 2020; Rollero et al., 2017). The combination of different omics methods is highly relevant to wine production and other food industries, as it can reveal the biological pathways underlying the formation of specific aroma compounds (Sirén et al., 2019). However, only a few studies have investigated the relationship between the microbial populations and sensory characteristics of wine (Perpetuini et al., 2022; Rossetti et al., 2023). In existing research, the integration of microbiome and volatile metabolome data has revealed that the spatial–temporal distribution of fungal and bacterial taxa is non-random and forms discrete “microbial niches” whose metabolic outputs are directly reflected in the wine volatile profile (Martins et al., 2024). Although the established principles of microbial niche dynamics and their metabolic consequences have been validated in grape-wine systems, it remains unclear whether these models apply to other fruit wine ecosystems, in which the distinct physicochemical properties of the fruit may select for unique microbial consortia and different competitive strategies.

Native to Southeast Asia and the Indo-China region, pomelo (*Citrus maxima* or *Citrus grandis*), a member of the *Rutaceae* family, has been garnering attention worldwide. Known for its fragrant aroma, delightful flavor, and impressive nutritional profile, this citrus fruit is also known for its medicinal properties (Goh et al., 2020; Zhang et al., 2011; Zhao et al., 2019; Methacanon et al., 2014). Jinggang honey pomelo, a premium quality fruit grown in Ji’an City, Jiangxi Province, has an exceptional nutritional profile. Compared with other pomelo varieties, Jinggang honey pomelo has higher concentrations of vitamin C, calcium, and magnesium (Sun et al., 2025). Jinggang honey pomelo wine is a fruit wine that has a unique citrus floral aroma, which may be caused by different microbial community compositions and ecological strategies compared with other wines.

Despite extensive studies on microbial succession in wine, the role of niche competition among microbial communities in aroma formation remains poorly understood. To test the hypothesis that niche competition between fungi and bacteria significantly influences aroma formation in pomelo wine, the present study combined high-throughput amplicon sequencing [16S/internal transcribed spacer (ITS)] to profile microbial succession using headspace solid phase microextraction (HS-SPME) coupled with gas chromatography–mass spectrometry (GC-MS) to track volatile compound dynamics. The present results extend the applicability of the microbial niche paradigm beyond grapes and provide an ecological blueprint for precise regulation of fruit wine flavor.

## 2 Materials and methods

### 2.1 Materials

Hydrochloric acid [HCl; 1 + 1 (v/v)], sodium hydroxide (NaOH) (200 g/L), NaOH (0.05 mol/L), copper sulfate (CuSO_4_;0.05 g/mL), glucose standard solution (2.5 g/L), methylene blue (10 g/L), phenolphthalein, and hexane (10 μg/mL) were purchased from Macklin (Shanghai, China). Sodium chloride (NaCl) and Seignette salt were purchased from SCR (Tianjing, China). Pectinase was purchased from Zhongchen Biotechnology Co., Ltd. (He’nan, China). Citric acid/sodium citrate was purchased from Yingxuan Industrial Co., Ltd. (Shandong, China). Fruit wine yeast was purchased from YADA (Hunan, China).

### 2.2 Sampling

In late October 2024, ripe Jinggang honey pomelos were picked from orchards in Ji’an, Jiangxi Province, China. Fully mature fruits, free from any signs of rot or mold, were selected for the fermentation experiment. The process was performed under controlled laboratory conditions, maintaining a steady room temperature of 20 °C throughout the process. The fruit pulp was crushed using a fruit pulper, and the crushed pulp was then transferred into a fermentation tank. After adding pectinase (4 g/L) to the fermentation tank, the Brix was adjusted to 22°Brix, and the pH was adjusted to 4.0. According to fruit wine yeast preparation instructions, half of the recommended amount of yeast (3 g/L) was weighed and placed in a small beaker containing a small amount of pure water at 30 °C. The yeast was placed in a water bath at 30 °C for activation, after which it was transferred to the fermentation tank and mixed thoroughly. The fermentation process was concluded after 14 days. The estimated alcohol content at the end of fermentation was 10 v/v% (semisweet type). Samples (*n* = 18) were taken at Days 0, 1, 4, 7, 10, and 14 of fermentation and frozen at −80 °C for subsequent analysis.

### 2.3 Physicochemical analysis

The following physicochemical parameters of the samples were determined: pH, Brix, total acid (TA), total sugar (TS), and alcohol content. The pH level was measured using a digital pH meter (PH-100, Shanghai, China). The TA content was determined via potentiometric titration and expressed as grams of tartaric acid per liter (g/L), and a pH of 8.2 served as the end point for titration. The total soluble solid (TSS) content was measured using a handheld Brix meter. The alcohol content was determined using an alcohol meter (CJM-091, China). The TS content was determined via direct titration and calculated using the following formula: TS = F × 1,000/[(V1/V2) × V3]; where TS denotes total sugar content (g/L); F (g) is the number of grams of glucose corresponding to 5 mL of each of Fehling’s solution (I and II); V1 (mL) is the volume of the sample taken; V2 (mL) is the volume of sample diluted; and V3 (mL) is the volume of the consumed sample.

### 2.4 Analysis of microbial diversity

Total genomic DNA samples were extracted using the OMEGA Soil DNA Kit (M5635-02) (Omega Bio-Tek, Norcross, GA, USA) according to the manufacturer’s instructions and stored at −20 °C until further analysis. The quantity and quality of the extracted DNAs were measured using a NanoDrop NC2000 spectrophotometer (Thermo Fisher Scientific, Waltham, MA, USA) and agarose gel electrophoresis, respectively. The hypervariable V3–V4 region of the bacterial 16S rRNA gene and the fungal ITS1 region were amplified and sequenced on an Illumina NovaSeq 6000 platform (Illumina, San Diego, CA, USA) with paired-end (PE) 250 bp sequencing strategy. The ITS1 region was amplified by the ITS5-1737F (5′-GGAAGTAAAAGTCGTAACAAGG-3′) and ITS2-2043R (5′-GCTGCGTTCTTCATCGATGC-3′) primers, and the V3–V4 regions of the 16S rRNA genes were amplified using the 338F (5′-ACTCCTACGGGAGGCAGCA-3′) and 806R (5′-GGACTACHVGGGTWTCTAAT-3′) primers (Li et al., 2023). The PCR amplification program for the 16S rRNA gene was as follows: initial denaturation at 98 °C for 5 min; 25 cycles of denaturation at 98 °C for 30 s, annealing at 52 °C for 30 s, and elongation at 72 °C for 45 s; and a final extension at 72 °C for 5 min. The PCR program for the ITS region was similar but with 30 cycles and an annealing temperature of 55 °C.

Microbiome bioinformatics was performed using QIIME2 (Bolyen et al., 2019), with slight modifications according to the official tutorials. Briefly, raw sequence data were demultiplexed using the demux plugin, followed by primer cutting using the cutadapt plugin. The sequences were quality-filtered, denoised, and merged, and chimeras were removed using the DADA2 plugin. Non-singleton amplicon sequence variants (ASVs) were aligned using mafft and used to construct a phylogeny using fasttree2. Taxonomic assignment was performed using the classify-sklearn naïve Bayes taxonomy classifier within the QIIME2 feature-classifier plugin. Bacterial ASVs were classified against the SILVA 16S rRNA gene database (Release 140), and fungal ASVs were classified against the UNITE ITS database (Release 9.0).

### 2.5 Analysis of volatile compounds

The volatile compounds in the samples were analyzed via HS-SPME coupled with GC-MS using the following parameters: fiber type, SPME arrow; incubation temperature, 60 °C; preheating time, 5 min; incubation time, 15 min; and desorption time, 5 min. The analysis was conducted using a gas chromatograph system (Agilent, California, USA) coupled with a mass spectrometer (Agilent, California, USA). A liquid sample (1 mL) was added to a headspace bottle and mixed with 2 mL of saturated NaCl solution and 20 μL of internal standard solution (3-hexanone-2,2,4,4-d4), and the sample was then extracted via HS-SPME coupled with GC–MS analysis. For the GC procedure, helium was the carrier gas, with a constant flow rate of 1.5 mL/min through the column. The initial temperature was maintained at 40 °C for 3.5 min, followed by a programmed increase to 100 °C at a rate of 10 °C/min, an increase to 180 °C at a rate of 7 °C/min, and an increase to 280 °C at a rate of 25 °C/min, where it was held for 5 min. The injection, transfer line, ion source, and quad temperatures were set at 250 °C, 250 °C, 230 °C, and 150 °C, respectively. The electron-impact mode was used with an energy of −70 eV. Mass spectrometry data were acquired by SIM. The NIST 17.0 Mass Spectral Library^1^ and Chroma TOF 4.3X software (Michigan-based LECO Corporation, USA) were used for raw peak extraction, baseline filtering, baseline correction, peak alignment, deconvolution analysis, peak identification, integration, and spectral matching. These procedures facilitated the qualitative and quantitative identification of volatile compounds.

### 2.6 Calculation of the relative odor activity value (rOAV)

The potential contribution of volatile compounds to the overall aroma profile was evaluated by calculating the rOAV for each compound using the following equation:


$$\mathrm{rOA}V_{i}=100\times \frac{C{\%}_{i}}{C{\%}_{\max}}\times \frac{T_{\max}}{T_{i}}\#$$

Where C%_max_ indicates the most abundant compound; T_max_ is the threshold; C%_i_ is the % content of a volatile compound; and Ti is the odor threshold in water.

A higher rOAV indicates a greater potential contribution of the compound to the aroma. Typically, compounds with an rOAV ≥ 1 are considered to have a direct impact on the aroma. In the present study, given the semi-quantitative nature of the concentration data, the rOAVs were used primarily for relative comparison and ranking of aroma compounds across different fermentation time points to identify the most impactful differential aroma compounds rather than for absolute quantitative assessment.

### 2.7 Data analysis

Statistical analysis was performed using SPSS 21.0 (IBM SPSS Inc., USA). To assess the richness and diversity of microorganisms within the samples, Chao1 index and Shannon index were calculated via R packages. Principal component analysis (PCA) was employed to assess microbial communities and visualize the distribution of volatile compound data. Analysis of similarity (ANOSIM) and permutational multivariate analysis of variance (PERMANOVA) assessed group differences using Bray–Curtis dissimilarity matrices. Orthogonal partial least squares-discriminant analysis (OPLS-DA) was performed using the “muma” R package (Mahadevan et al., 2008). Origin 2021 Pro (OriginLab Corp., Northampton, MA, USA) was used to generate histograms. Correlation analysis between physicochemical property variables and core microbiota in pomelo fermentation was performed via redundancy analysis (RDA) using the “vegan” R package. The differential volatile compounds were analyzed using the Kyoto Encyclopedia of Genes and Genomes (KEGG) database to identify related metabolic pathways. The network analysis was performed using R and visualized by Gephi (v0.9.5).

## 3 Results and discussion

### 3.1 Changes in physicochemical properties during fermentation

The changes in key physicochemical properties during fermentation are summarized in Table 1, and the fermentation kinetics curve based on TSS consumption and alcohol production are depicted in Supplementary Figure S1. On the basis of the kinetic profiles, which reflect microbial growth and metabolic activity, the fermentation process was divided into the following five distinct stages: the initial stage (GR-0d to GR-1d), the logarithmic stage (GR-1d to GR-4d), the late-exponential stage (GR-4d to GR-7d), the early-stationary stage (GR-7d to GR-10d), and the late-stationary stage (GR-10d to GR-14d). The refined division provided a robust framework for analyzing the subsequent microbial succession and metabolite changes.

**Table 1:** Changes in physicochemical properties during pomelo wine fermentation.

Physicochemical indexes	Fermentation procedures					
	0 days	1 days	4 days	7 days	10 days	14 days
pH	4.000 ± 0.003^a^	3.753 ± 0.003^c^	3.783 ± 0.003^c^	3.847 ± 0.007^b^	3.830 ± 0.006^b^	3.840 ± 0.006^b^
TA (g/L)	5.270 ± 0.100^d^	6.670 ± 0.100^c^	8.487 ± 0.017^b^	8.643 ± 0.027^a^	8.673 ± 0.027^a^	8.773 ± 0.019^a^
TS (g/L)	90.217 ± 0.033^c^	71.933 ± 1.821^e^	86.513 ± 0.018^d^	97.790 ± 1.700^b^	104.867 ± 0.982^a^	105.073 ± 0.907^a^
TSS (%)	20.000 ± 0.033^a^	18.333 ± 0.167^b^	13.333 ± 0.167^c^	10.333 ± 0.167^d^	8.167 ± 0.167^e^	7.667 ± 0.167^e^
Alcohol (v/v, %)	n.d.	n.d.	4.200 ± 0.058^d^	6.167 ± 0.033^c^	9.133 ± 0.033^b^	10.033 ± 0.088^a^

The values are presented as the mean ± standard error (*n* = 3); a–e, different letters indicate significant differences (*p* < 0.05, one-way ANOVA); n.d., not detected.

At the start of fermentation (GR-0d), the solution had a pH of 4.000, TA content of 5.270 g/L (expressed as tartaric acid), a TS content of 90.217 g/L, and TSS content of of 20.000%. As the microbial community begins to flourish, enzymes produced by these microorganisms facilitate the breakdown of carbohydrates, proteins, and fats through enzymatic reactions (Carpena et al., 2020). During fermentation, a series of enzymatic processes—combined with adsorption and flocculation—break down complex organic compounds into simpler molecules, including various acids (such as lactic, tartaric, malic, succinic, citric, and acetic acids) and polymeric metabolites (Yuan et al., 2022). These biochemical byproducts subsequently alter key winemaking parameters. By GR-14d, notable shifts were observed; the pH and TSS contents decreased to 3.840 and 7.667%, respectively, whereas the TA and TS contents increased to 8.773 and 105.073 g/L, respectively. Moreover, there was no alcohol content observed on Day 1, but it gradually increased to 10.033% v/v. Under the action of yeast, glucose, fructose, and other sugars are converted into pyruvate through the glycolysis pathway. Pyruvate undergoes decarboxylation via the pyruvate decarboxylase enzyme, yielding acetaldehyde as an intermediate. This compound is subsequently reduced to ethanol through alcohol dehydrogenase (ADH) activity, resulting in a steady accumulation of alcohol over time (Adebami et al., 2022). In addition to ethanol and carbon dioxide, the fermentation process produces other byproducts, such as glycerol, higher alcohols (e.g., isoamyl alcohol), and esters. These substances have a substantial impact on the flavor of fruit wine (Styger et al., 2011).

### 3.2 Microbial succession

**Figure 1:**
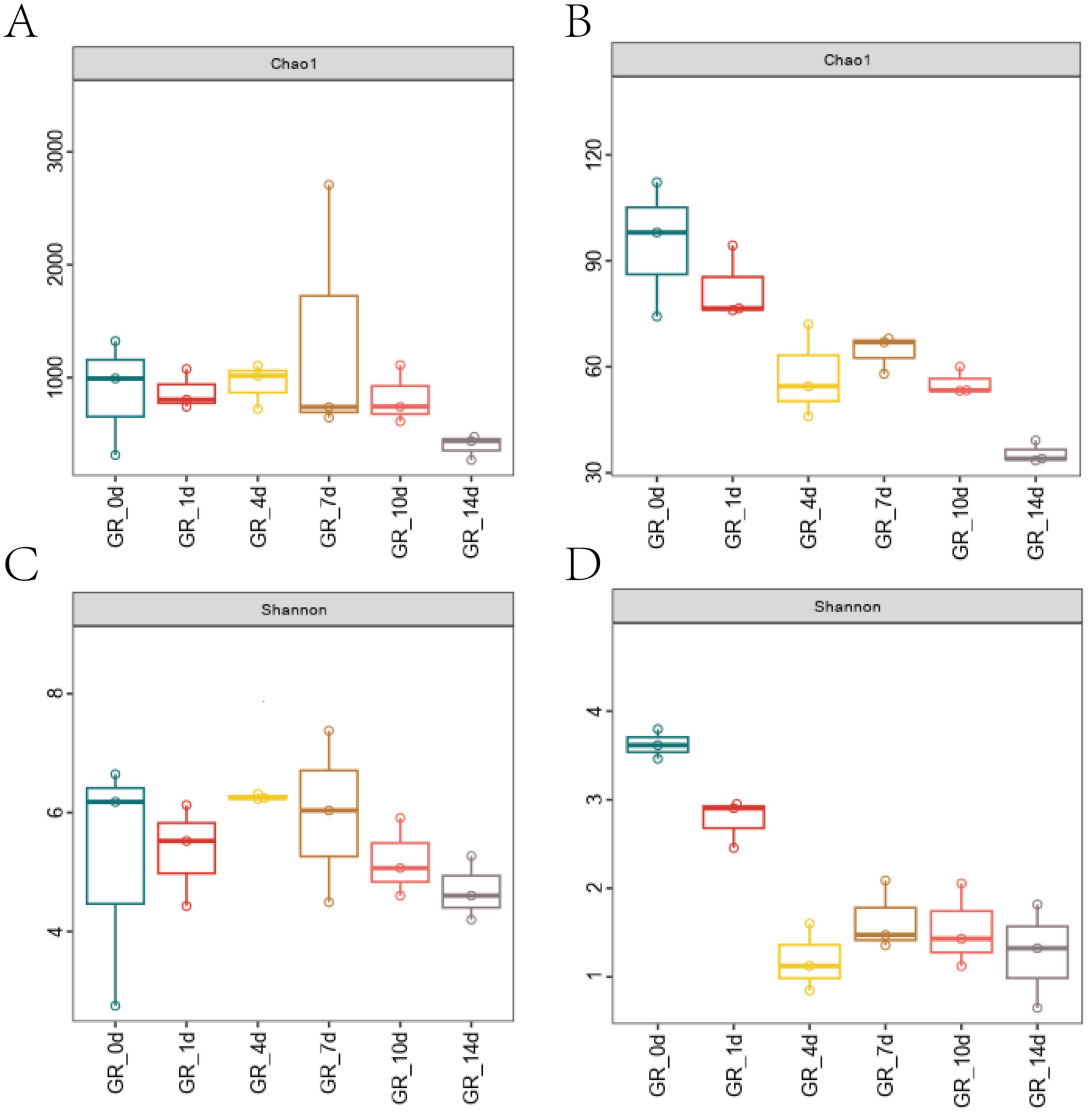
Richness and diversity of microorganisms. **(A,B)** Bacterial and fungi Chao indices. **(C,D)** Bacterial and fungi Shannon indices.

Analysis of the V3–V4 region of the 16S rRNA gene across 18 samples generated 1,530,938 quality reads using QIIME2. The samples averaged 85,052 sequences, and the denoising process using the DADA2 plugin generated 8,857 bacterial ASVs. High-throughput fungal sequencing yielded 2,093,784 quality reads, averaging 116,321 per sample, and the denoising process identified 536 fungal ASVs. Figure 1 shows the microbial diversity and richness according to the Shannon and Chao indices. The diversity of fungi and bacteria in fermentation typically decreases over time, especially during the initial stages (GR-0d to GR-1d) when conditions, such as temperature, pH, and sugar, promote yeast growth. Non-*Saccharomyces cerevisiae* usually dominates at this stage. As fermentation progresses, the sugar content in the fermentation liquid is gradually consumed, and the alcohol concentration starts to increase, exerting a certain inhibitory effect on non-*S. cerevisiae* growth. At this point, *S. cerevisiae* began to dominate (Fleet, 2003). In early-stationary and late-stationary fermentation stages, alcohol levels increase, nutrient levels decrease, and yeast growth is stunted, causing yeast cell death, resulting in a gradual decline in the richness and diversity. For bacteria, in the logarithmic stages of fermentation, bacterial genera, such as *Acetobacter* and *Lactobacillus*, begin to appear and gradually increase, exhibiting an upward trend from GR-1d to GR-4d. As fermentation continues, *Lactobacillus* becomes the dominant species, becoming active in the early-stationary phase of fermentation and conducting malolactic fermentation, which converts malic acid to lactic acid, thereby reducing the acidity of wine. In the early-stationary and late-stationary stages of fermentation, the nutrients in the fermentation liquid decrease, restricting bacterial growth and leading to a decline in richness and diversity.

**Figure 2:**
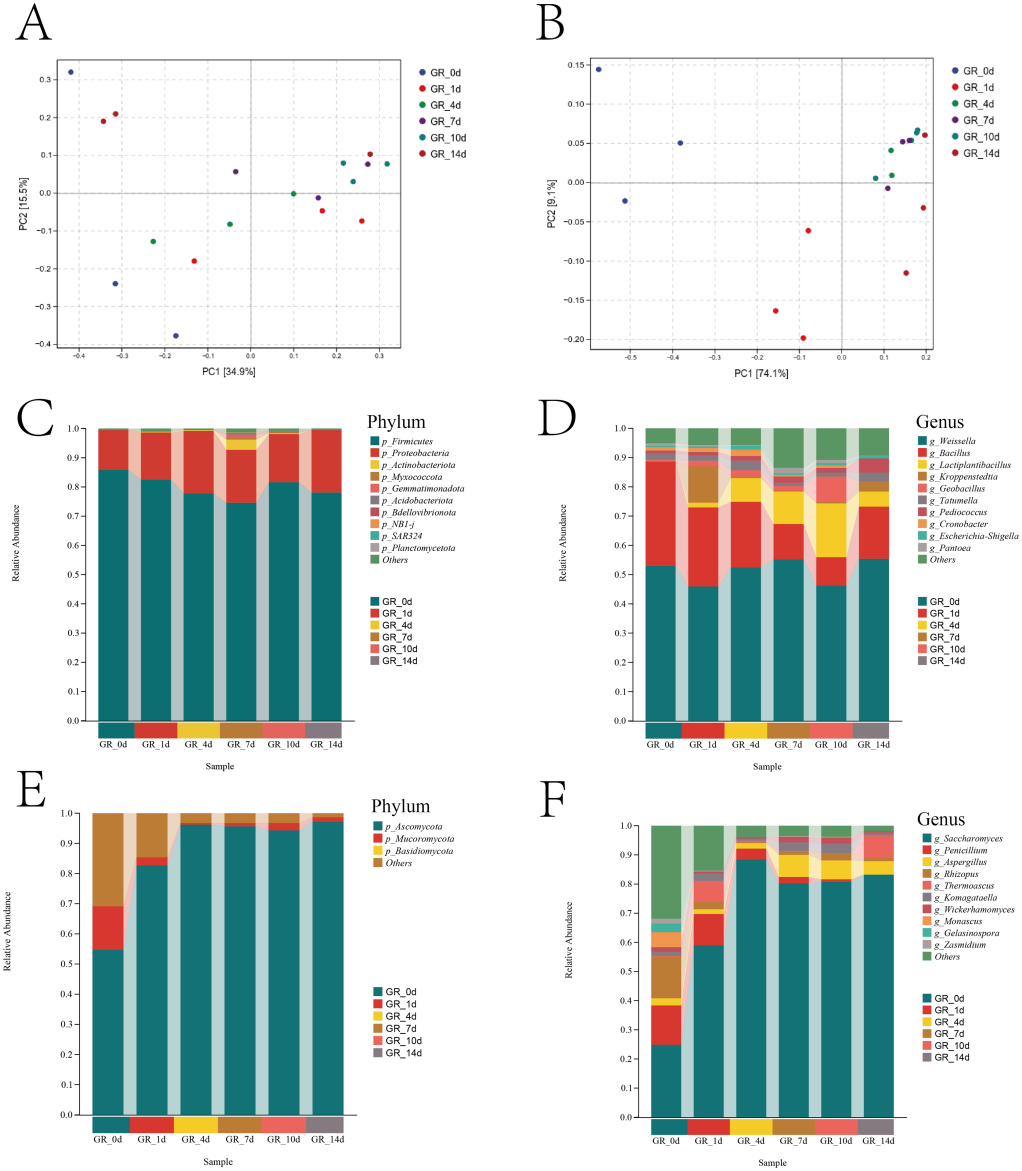
Microbial communities shift in pomelo wine fermentation. PCA revealed the evolving bacterial **(A)** and fungal **(B)** communities across fermentation stages, calculated using Bray–Curtis distance. Bacterial community succession at the phylum **(C)** and genus **(D)** levels. Fungal community succession at the phylum **(E)** and genus **(F)** levels (ANOVA, *p* < 0.05).

PERMANOVA revealed significant differences in bacterial (*R*^2^ = 0.4334, *p* = 0.011) and fungal (*R*^2^ = 0.8135, *p* = 0.001) communities. PCA showed distinct microbial community separation and differentiation at Days 0, 1, 4, 7, 10, and 14. The variation in the fungal communities was mostly explained (83.2%) by the first two axes, while the variation in the bacterial communities was mainly explained (50.4%) by the remaining axes (Figures 2A,B).

The bacterial community underwent a clear ecological succession, transitioning from a diverse initial state to a community dominated by acid-tolerant lactic acid bacteria, a process largely driven by metabolic acidification (Figures 2C,D). At the initial stage of fermentation (GR-0d to GR-1d), the community was highly diverse, containing substantial proportions of *Weissella*, *Bacillus*, *Lactiplantibacillus*, *Kroppenstedtia*, and *Geobacillus*. Lactic acid bacteria, represented primarily by *Weissella*, *Lactiplantibacillus*, and *Pediococcus*, constituted 54.12% of the total bacterial population. The logarithmic stage of fermentation (GR-1d to GR-4d) was characterized by the proliferation of lactic acid bacteria, as their proportion steadily increased as they began to utilize available nutrients. A pivotal shift occurred post-GR-4d. At this stage, lactic acid bacteria, particularly *Weissella*, increased their glycolytic metabolism by upregulating key enzymes, such as glucokinase, dehydrogenase, and lactate dehydrogenase. This metabolic burst initiated malolactic fermentation and led to a substantial decrease in the pH of the fermentation broth (Tian et al., 2021; Wang et al., 2021; Wang et al., 2022). This acidification event acted as a major environmental filter, shaping the community structure in the subsequent phase (GR-4d to GR-14d). The acidic stress disrupted the microenvironmental equilibrium, making it inhospitable for acid-sensitive genera. As a result, the abundance of *Bacillus* and the opportunistic pathogen *Cronobacter* decreased from 2.19% to 0.01%. Consequently, the community became dominated by acid-tolerant lactic acid bacteria. By the end of fermentation (GR-14d), the lactic acid bacteria population further increased and stabilized at 65.65%.

Throughout this process, *Weissella* remained the consistently dominant genus (46.05%–55.44%), playing a crucial role in acid production and in synthesizing key volatile compounds that contribute to the aroma of the product (Tan et al., 2024). The enrichment of *Lactiplantibacillus* and *Pediococcus* further enhanced flavor complexity and product safety (Yan et al., 2024; Todorov et al., 2023). Thus, the bacterial succession was characterized by lactic acid bacteria-driven acidification, which in turn reinforced lactic acid bacteria dominance, creating a self-sustaining cycle that shaped the final microbial landscape and metabolic output.

In contrast to the bacterial community, the fungal community exhibited a more rapid and dramatic succession, marked by the competitive exclusion of non-fermentative fungi by fermentative yeasts (Figures 2E,F). The pre-fermentation community (GR-0d) was a complex assemblage dominated by non-fermentative fungi, including filamentous fungi, such as *Penicillium* (13.33%) and *Rhizopus* (14.25%), alongside other non-*S. cerevisiae* yeasts (31.96%). This composition decreased to 0% after fermentation began (GR-1d). Fermentative yeasts, notably *Saccharomyces* and *Komagataella*, began their exponential growth. The competitive dominance of *S. cerevisiae*, evidenced by its superior fermentation rate, ethanol yield, and tolerance to anaerobic conditions, created a selective pressure that non-*S. cerevisiae* species could not withstand (Liu et al., 2022). The logarithmic stage of fermentation represented the critical transition window. During this short span, the previously abundant filamentous fungi and non-fermentative yeasts were rapidly outcompeted, with some genera, such as *Monascus*, *Gelasinospora*, and *Zasmidium*, decreasing to undetectable levels. From GR-4d onwards, the fungal community was dominated by a few fermentative yeasts. *Saccharomyces* emerged as the dominant genus, with a massive surge in relative abundance from 59.07% (GR-0d) to a peak of 83.18% (GR-14d). This establishment of a low-diversity, high-abundance fermentative community, particularly the enrichment of aroma-producing fungi at GR-4d, facilitated the development of a distinctive and complex aroma profile in the final product through synergistic metabolic interactions. The succession illustrated a classic example of how a specialized, highly adapted organism (*S. cerevisiae*) can restructure an entire microbial community through resource competition and stress tolerance.

### 3.3 Keystone taxa in the co-occurrence network of pomelo wine fermentation

**Figure 3:**
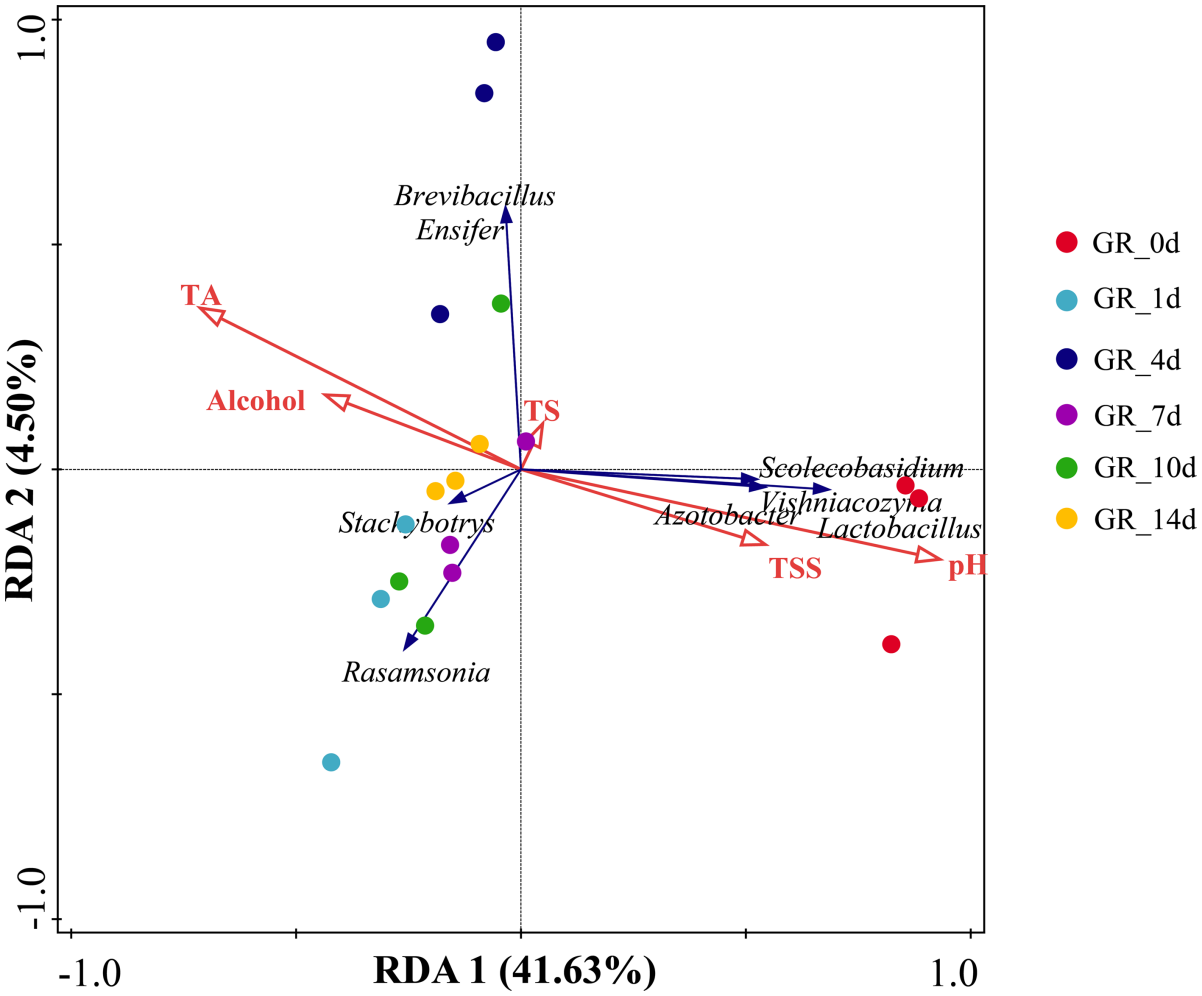
Co-occurrence network of bacteria and fungi. Fungi (red nodes) and bacteria (green nodes) showed positive (red) and negative (blue) correlations. Node size indicates connectivity strength—larger nodes signify higher connectivity. Microbial taxa (*Scolecobasidium*, *Vishniacozyma*, *Rasamsonia*, *Azotobacter*, *Stachybotrys*, *Lactobacillus*, *Brevibacillus*, and *Ensifer*) with higher nodes are marked.

A co-occurrence network was constructed based on the top 30 bacteria and fungi according to relative abundance. The keystone taxa (key nodes) in the network were identified based on their connectivity greater than or equal to 3. Eight key nodes occupying central positions in the network structure were identified, and these nodes may play an important role in maintaining the stability of the fermentation microbial community. The key nodes included *Scolecobasidium*, *Vishniacozyma*, *Rasamsonia*, *Azotobacter*, *Stachybotrys*, *Lactobacillus*, *Brevibacillus*, and *Ensifer*, all of which showed positive correlations with each other (Figure 3).

Among the fungal taxa, *Vishniacozyma* (connectivity = 7) exhibited the strongest connectivity within the network, suggesting that it may play a pivotal role in the microbial interaction network during the fermentation process. Previous studies have reported that *Vishniacozyma* has fermentative ability, indicating that this yeast can produce various volatile compounds, including higher alcohols, esters, and organic acids, which significantly influence the aromatic and taste characteristics of wine (Reiter et al., 2021). Similarly, other highly connected species also demonstrated adaptability to the fermentation environment. For example, *Rasamsonia* (connectivity = 4), a thermotolerant fungal genus, has been reported to enhance fruit wine flavor when co-fermented with other microorganisms (Chen et al., 2020). The central network position of *Lactobacillus* (connectivity = 3), a common lactic acid bacterium in fermented products, related to its acidification function during fermentation (Houbraken et al., 2012).

Some species, such as *Azotobacter* (connectivity = 5) and *Stachybotrys* (connectivity = 4), also showed high connectivity in the network. *Stachybotrys* produces a diverse array of secondary metabolites and may participate in the metabolism of polyphenolic and flavonoid compounds (Baars et al., 2016). *Azotobacter*, a Gram-negative bacterium with nitrogen-fixing capabilities, provides an additional nitrogen source, thereby promoting the growth and metabolism of yeast and enhancing fermentation efficiency (Velić et al., 2018). These highly connected species may influence the dynamic balance of the fermentation microenvironment through complex ecological interactions.

Network connectivity provides information about the centrality of species within the community structure. These highly connected species may affect the fermentation process through direct metabolic interactions or indirect environmental modulation.

### 3.4 Correlation between Core microbiota and physicochemical properties

**Figure 4:**
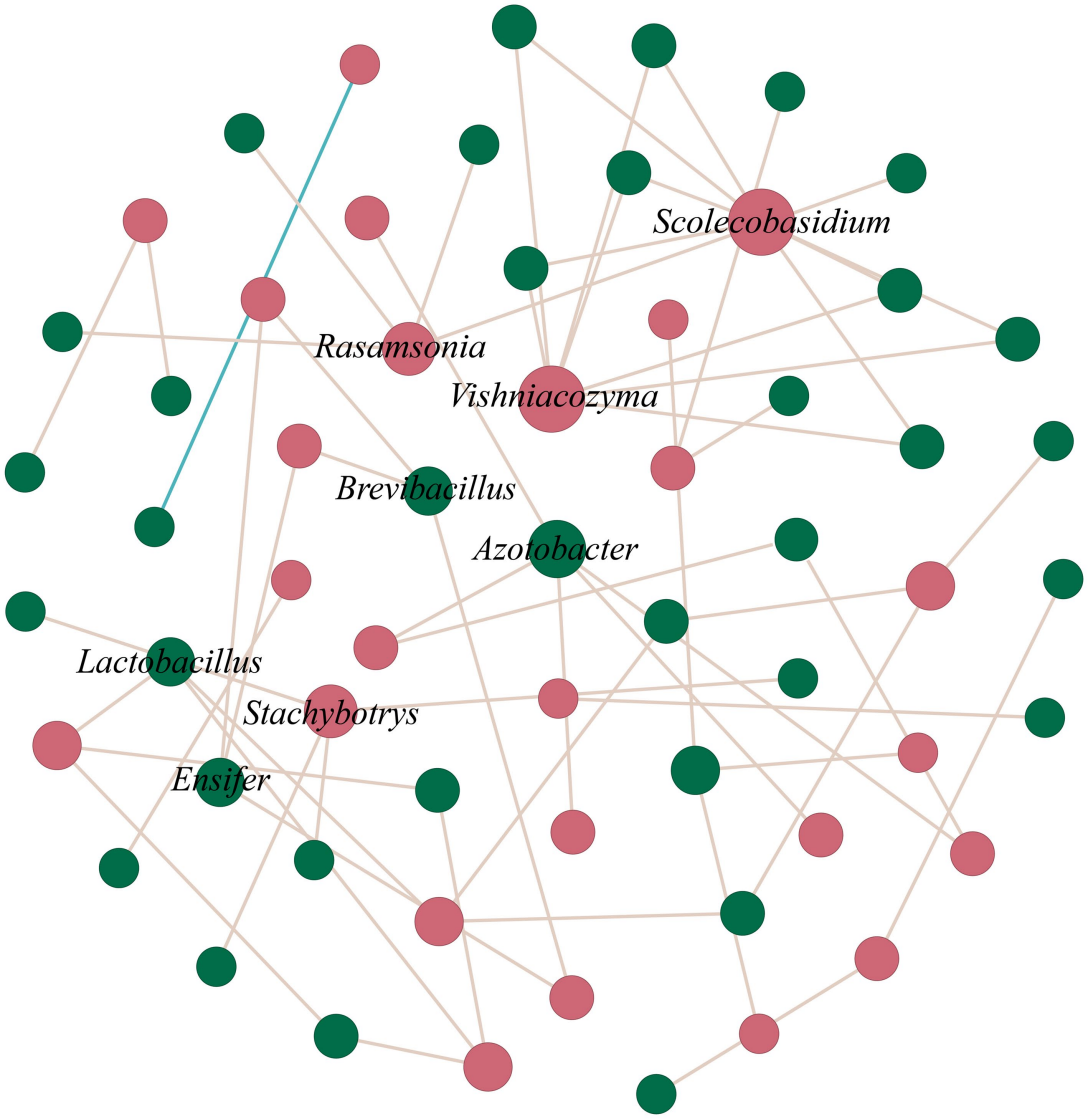
Correlation between core microbiota and oenological parameters. RDA revealed the relationship between key microbial communities and their corresponding physicochemical characteristics. Red arrows denote the different oenological parameter variables, and blue arrows denote the core microbiota (*Scolecobasidium, Vishniacozyma, Rasamsonia, Azotobacter, Stachybotrys, Lactobacillus, Brevibacillus*, and *Ensifer*). Circles with different colors represent samples from different time periods.

The formation, growth, and replacement of the key microflora are pivotal in influencing the key characteristics of wine, such as pH, TS, TA, TSS, and alcohol levels (Virdis et al., 2021). Redundancy analysis (RDA) was performed to explore the link between these parameters and the microflora over the course of fermentation (Figure 4). The primary canonical axis explained 41.63% of the variance of the core microbiota. Core microbiota evolution emerged as a significant factor influencing changes in oenological traits (Figure 4). *Brevibacillus*, *Ensifer*, and *Stachybotrys* showed robust association with alcohol and TA levels in the fermentation process but only slight association with pH and TSS levels, suggesting that these genera are conducive to generating alcohol and TA during fermentation (Peng et al., 2021; Suribabu et al., 2014; Tsegay et al., 2018). Conversely, *Scolecobasidium*, *Vishniacozyma*, *Azotobacter*, and *Lactobacillus* were closely associated with pH levels and increased TSS contents. These microorganisms are particularly sensitive to acidic conditions and may regulate microbial competition through acidity, leading to changes in their population and overall abundance. As the fermentation process progresses, the dominant microbial communities evolve, allowing them to adjust to shifting environmental conditions while maintaining their metabolic activity. This dynamic adaptation ensures the survival of these microbial communities and preserves their crucial role in generating distinctive aromatic compounds (Virdis et al., 2021). Therefore, throughout the fermentation process, the competition for the ecological niches of these microorganisms plays an important role in the formation of the complex flavor of pomelo wine.

### 3.5 Dynamic changes in volatile compounds during pomelo wine fermentation

**Figure 5:**
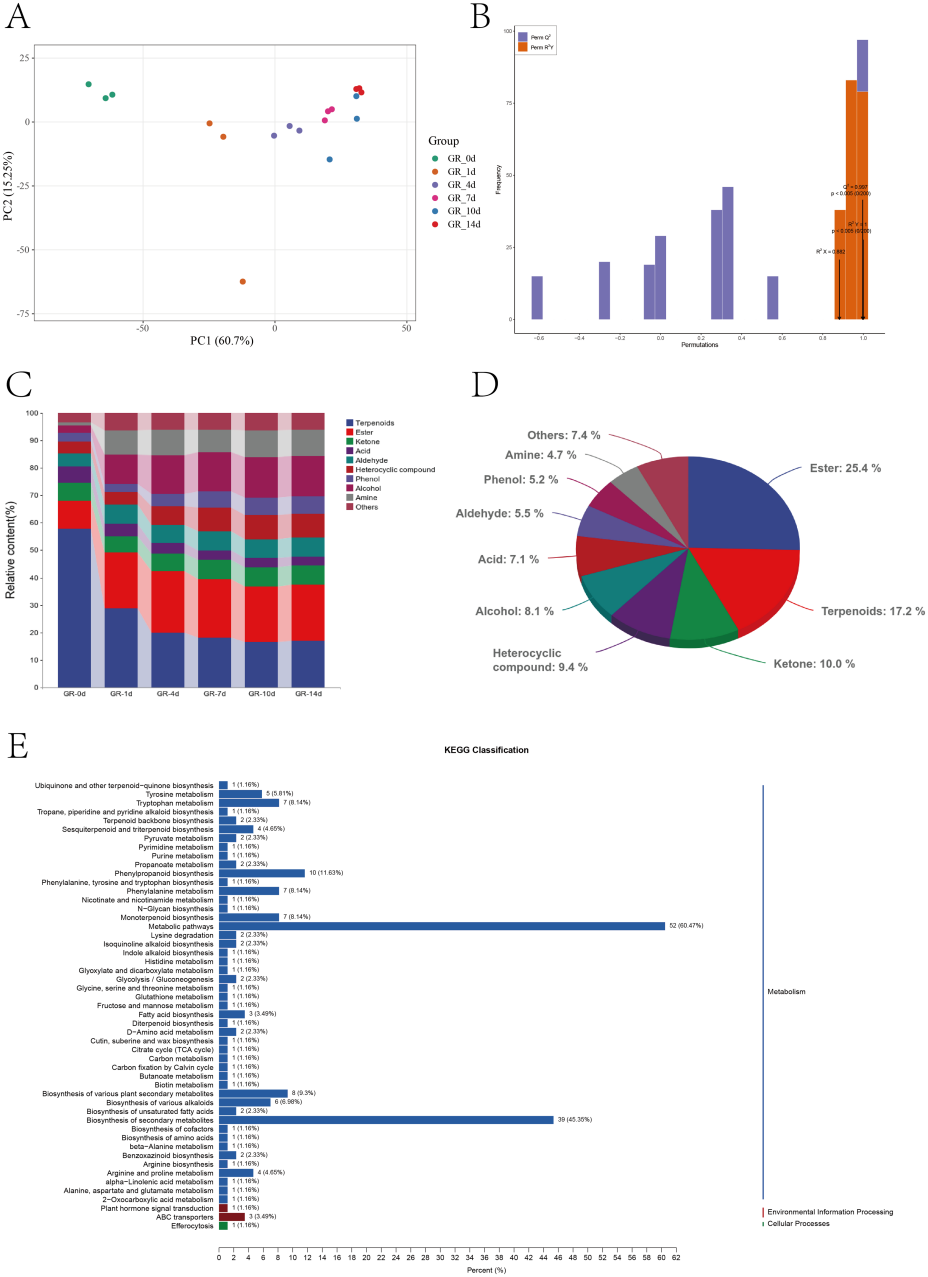
Analysis of volatile compound evolution during pomelo wine fermentation. **(A)** PCA score scatter plot (all). **(B)** OPLS-DA score plot comparing fermentation stages (GR-0d vs. GR-14d). **(C)** Distribution profile of volatile compound classes (terpenoids, esters, ketones, acids, aldehydes, heterocyclics, phenols, alcohols, and amines) across fermentation time—highlighting the top nine most abundant compounds by relative percentage. **(D)** Abundance stacking map showing dynamic changes in volatile compounds. **(E)** KEGG metabolite statistics.

In total, 2,012 volatile compounds, including esters, terpenoids, ketones, heterocyclic compounds, alcohols, acids, aldehydes, amines, phenols, and hydrocarbons, were detected in pomelo wine fermentation via HS-SPME coupled with GC-MS (Supplementary Table S1). Esters constituted a large portion of the volatile compounds, with 408 types, followed terpenoids, with 370 types. PCA and discriminant analysis of volatile compounds effectively differentiated the samples that were fermented at various stages. Principal component (PC) 1 and PC2 explained 72.43% of the overall variance, with PC1 contributing 58.19% of the variance and PC2 contributing 14.24% of the variance (Figure 5A). PCA revealed notable variations in the volatile compound profiles. In addition, OPLS-DA analysis was conducted to distinguish the differential volatile compounds in the GR-0d and GR-14d fermentation groups (*R*^2^X = 0.882, *R*^2^Y = 1, *Q*^2^ = 0.997) (Figure 5B).

Among the rich compounds present in pomelo, terpenoids and acids are an important source of the unique aroma and flavor of pomelo. The relative content of terpenoids decreased by 40.66% and that of acids decreased by 2.59% (Figure 5C). This reduction may be related mainly to microbial niche transformation. During fermentation, enzymes produced by microorganisms, such as aldo–keto reductases (AKRs), may catalyze the conversion of terpenoids into other substances, thereby reducing their relative content (Zeng et al., 2025). Apart from these factors, microbial metabolism and the loss of volatile compounds also play a role. Yeasts and other microorganisms may utilize terpenoids as a carbon or energy source, leading to a decrease in their content (Huacachino et al., 2024). During fermentation, yeasts consume organic acids present in pomelo wine as a carbon source, converting them into other substances. For example, *S. cerevisiae* metabolizes malic acid into ethanol and carbon dioxide (Roberts et al., 2024).

The relative content of esters markedly increased (Figure 5C) from 10.26% in GR-0d to 20.28% in GR-14d. However, 47 ester compounds, including pentanoic acid phenylmethyl ester, cyclohexanol-1-methyl-4-(1-methylethylidene)-acetate, butanoic acid-2-methyl-phenylmethyl ester, carbonic acid phenyl propyl ester, 4-hexen-1-ol-acetate, and ethyl mandelate, gradually degraded during fermentation, potentially resulting from enzymatic hydrolysis reaction or volatilization loss (Vion et al., 2024; Ling et al., 2025). As the fermentation time prolonged, the ester levels in pomelo wine sharply increased, with some compounds increasing by more than 1,000 times [variable importance in projection (VIP) > 1]. Key players in this aromatic transformation included ethyl trans-4-decenoate, which emerged as the dominant volatile, alongside other notable esters, such as decanoic acid ethyl ester, dodecanoic acid ethyl ester, butanoic acid decyl ester, 2-heptanol acetate, butanoic acid 5-hexenyl ester, l-tyrosine methyl ester, and benzeneacetic acid methyl ester. These compounds collectively contributed to the evolving flavor profile of the wine during the fermentation process.

Ketones and aldehydes, derived from alcohol oxidation and carbohydrate breakdown, increased by 0.45 and 2.1%, respectively (Figure 5C). At GR-14d, the levels of differential volatile compounds—2-methyl-3(2-furyl) acrolein, 2,4-dioxabicyclo-hept-6-en-3-one- 1,5-dichloro-6,7-dimethyl, and xanthone—increased by several 1,000 times compared with those at GR-0d (Supplementary Table S2). However, the total proportion of these volatile compounds was relatively insignificant, likely due to the presence of alcohol-ketone reductases (AKRs) in microorganisms, enzymes that transform carbonyl compounds into their corresponding alcohols by reducing the carbonyl group to a hydroxyl group (Sumby et al., 2013).

Carbohydrates are the main source of alcohols, which are the precursors of aging esters, in fruit juice. Carbohydrates are converted during fermentation through the central carbon metabolic pathway and amino acid decarboxylation process that occur in yeast and bacterial cells. The main enzymes responsible for alcohol synthesis are alcohol dehydrogenases (ADHs), which promote the conversion of carbohydrates into alcohols (Penning, 2015). The content of ADHs increased from 2.72 to 14.74%, and ethanol-2-(2-butoxyethoxy) and ethene-1,1′-[oxybis(2,1-ethanediyloxy)]bis emerged as the dominant alcohols during pomelo wine fermentation (Figure 5C; Supplementary Table S2).

Heterocyclic compounds, phenols, and amines markedly influence the aroma and flavor of pomelo wine (Qian et al., 2019). The relative contents of heterocyclic compounds, phenols, and amines increased by 4.38%, 3.05%, and 8.52%, respectively. The most significant shifts in the relative content during pomelo wine fermentation were observed in several volatile compounds, namely, pyrazine-2,3-diethyl-5-methyl, phenol-3-ethyl, and benzene carbothioamide, which were identified as key differentiators (Figure 5C; Supplementary Table S2).

A comprehensive analysis identified 1,044 differential volatile compounds (VIP > 1, fold change ≥ 2 or ≤ 0.5, *p* < 0.05) when comparing the GR-0d and GR-14d samples (Supplementary Table S2). These compounds were classified into 14 broad categories—9 of which were predominant—including esters (266 types), terpenoids (180 types), ketones (105 types), heterocyclic compounds (98 types), alcohols (82 types), acids (74 types), aldehydes (58 types), phenols (54 types), amines (49 types), and others (78 types) (Figure 5D).

KEGG analysis was performed on the differential volatile compounds, and metabolic pathway information was obtained for the identified metabolites by comparison with the KEGG database (Figure 5E). In total, 51 metabolic pathways belonging to 3 categories were involved in the fermentation of pomelo wine. The main pathways included metabolic pathways (52 metabolites), biosynthesis of secondary metabolites (39 metabolites), phenylpropanoid biosynthesis (10 metabolites), biosynthesis of various plant secondary metabolites (8 metabolites), phenylalanine metabolism (7 metabolites), monoterpenoid biosynthesis (7 metabolites), tryptophan metabolism (7 metabolites), biosynthesis of various alkaloids (6 metabolites), and tyrosine metabolism (5 metabolites).

### 3.6 rOAV analysis of volatile compounds during pomelo wine fermentation

To assess the potential contribution of volatile compounds to the overall aroma, the rOAV was utilized as a screening tool. The rOAV is calculated based on semi-quantitative measurement data of volatile compounds. In this context, the primary value of the rOAV lies in the relative ranking and comparison of aroma impact across fermentation time points, rather than in providing absolute quantitative measures. In the present study, a higher rOAV suggested a greater potential contribution of a compound to the evolving aroma profile of the product.

**Figure 6:**
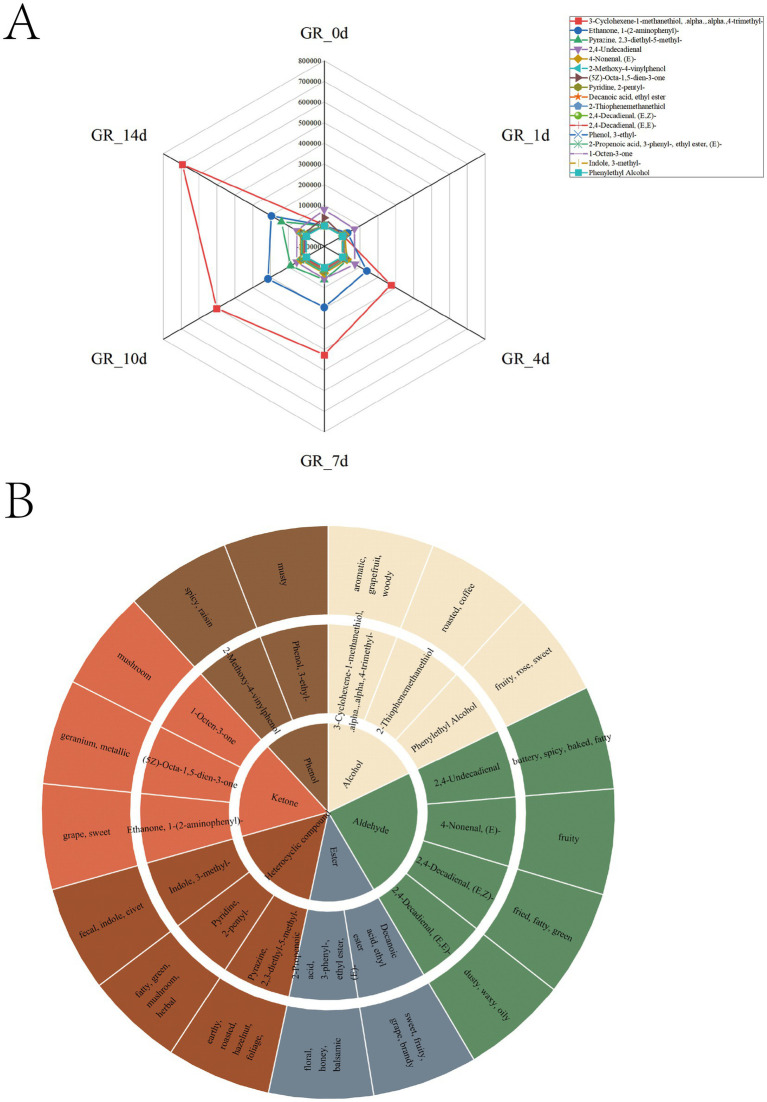
rOAV analysis of volatile compounds. **(A)** Fluctuations in the six dominant volatile categories (esters, ketones, aldehydes, heterocyclics, phenols, and alcohols) and their associated secondary metabolites (17 identified types) at different fermentation times (GR-0d to GR-14d). **(B)** Classification and aroma contributions of 17 metabolites.

In the present study, 238 aroma compounds were selected on the basis of their odor profiles and corresponding rOAV values (Supplementary Table S2). Among these, 17 compounds were considered key differential aroma compounds characterized by rOAVs exceeding 1,000, VIP scores greater than 1, fold changes greater than 2 or less than 0.5, and *p*-values less than 0.05 (Figures 6A,B; Supplementary Table S2). The main contributing compounds highlighting the aroma of pomelo in terms of fruity,” “green,” and “sweet” (GR-0d) were as follows: 2,4-undecadienal (rOAV = 78034.43); (5Z)-octa-1,5-dien-3-one (rOAV = 38319.38); 3-cyclohexene-1-methanethiol,alpha,alpha,4-trimethyl- (rOAV = 4500.32); ethanone, 1-(2-aminophenyl)- (rOAV = 904.36); 2-propenoic acid, 3-phenyl-, ethyl ester, (E)- (rOAV = 695.48); 2-thiophenemethanethiol (rOAV = 446.43); 1-octen-3-one (rOAV = 223.96); 4-nonenal, (E)- (rOAV = 138.74); pyrazine, 2,3-diethyl-5-methyl- (rOAV = 133.13); 2-methoxy-4-vinylphenol (rOAV = 116.36); indole, 3-methyl- (rOAV = 92.51); pyridine, 2-pentyl- (rOAV = 26.32); 2,4-decadienal, (E,Z)- (rOAV = 10.15); 2,4-decadienal, (E,E)- (rOAV = 10.15); phenol, 3-ethyl- (rOAV = 7.34); phenylethyl alcohol (rOAV = 6.82); and decanoic acid, ethyl ester (rOAV = 6.19) (Figures 6A,B; Supplementary Table S2). As fermentation extended over a longer period, both environmental conditions and microbial activity led to notable shifts in aroma profiles. For example, the aromas described as “fruity,” “sweet,” and “green” became more prominent after fermentation (GR-4d), blending with “grape” and “spicy” notes. Moreover, several qualities, such as “buttery,” “baked,” “fatty,” “metallic,” and “spicy” gradually faded throughout the fermentation process. In addition, the “aromatic” and “woody” aromas showed the most dramatic increase in intensity throughout fermentation. Furthermore, 4-nonenal, (E)- contributed mainly to the “fruity” aroma and was upregulated by 222.51-fold (rOAV = 30868.62) in the GR-14d sample (Figures 6A,B; Supplementary Table S2).

### 3.7 Correlation between the key microbiota and key differential aromas

Contextualization of the present findings within the broader scope of citrus wine fermentations revealed both convergent and divergent patterns. The successional dynamics observed in the present pomelo wine, characterized by the early diversity of fungi followed by the dominance of *S. cerevisiae*, and the persistent dominance of *Weissella* and *Lactiplantibacillus* among bacteria, align with findings in mandarin orange wine fermentations (Li et al., 2025; Wen et al., 2025; Hu et al., 2020). Similarly, the marked increase in ester compounds, particularly ethyl esters (such as decanoic acid ethyl ester) is a common driver of fruity aroma development in many fermented citrus beverages (Guo et al., 2025). However, the present study revealed potential distinctive features of pomelo wine. The high rOAVs of certain aldehydes, such as 2,4-undecadienal and 4-nonenal, (E)-, and their significant contribution to the “fruity” and “green” aroma profile are more pronounced than those reported in orange or lemon wines (Wang et al., 2024). These findings suggested that the unique chemical composition of pomelo may select for a specific microbial consortium or support metabolic pathways that differentially amplify these volatile compounds, thereby contributing to the characteristic aroma signature of pomelo wine.

**Figure 7:**
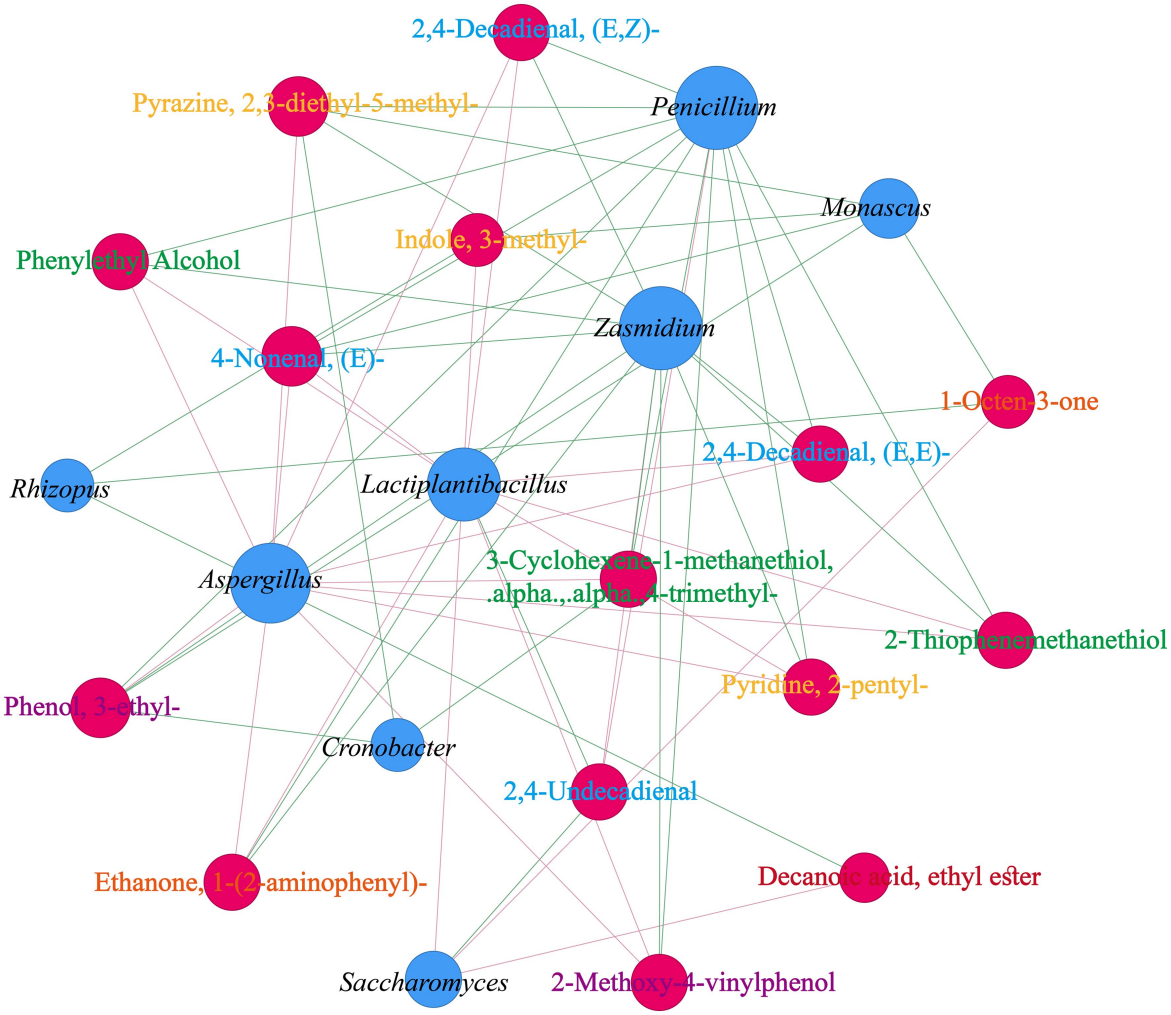
Relationship network between the core microbiota and key differential aroma compounds (Pearson; *R* > 0.6, *p* < 0.05). Red nodes show key differential aroma compounds (green, alcohol; orange, ketone; blue, aldehyde; red, ester; yellow, heterocyclic compound; purple, phenol), and blue nodes show the core microbiota. Pink and green lines signify correlations. Node size reflects connectivity.

To directly investigate the core functional microbiota driving the formation of characteristic aroma profiles in pomelo wine, a targeted correlation network was constructed. The core microbiota were derived from the 8 keystone taxa identified via the co-occurrence network analysis in Section 3.3, along with the top 20 fungal and bacterial species with the highest relative abundance throughout the fermentation process. These species, occupying central positions in the community structure or exhibiting high abundance, are most likely to exert a dominant influence on the metabolic functions of the system. The key differential aroma compounds were selected from the 17 volatile compounds screened by rOAV in Section 3.6, which represent the most prominent candidates responsible for shaping the final characteristic aroma of pomelo wine. Pearson’s correlation analysis (*R >* 0.6, *p* < 0.05) was used to map the relationships between the core microbiota and key differential aromas (Figure 7). This network shed light on how specific microbiota influence the development of distinct aromas throughout the fermentation of pomelo wine. *Penicillium*, *Aspergillus*, *Lactiplantibacillus*, *Zasmidium*, *Monascus*, and *Saccharomyces* showed a strong association with the key differential aromas. Among them, *Lactiplantibacillus* exhibited increased relative abundance (0.00%–18.25%) during the GR-0d to GR-10d period. The following key differential aromas gradually increased during the GR-0d to GR-10d period and were positively associated with *Lactiplantibacillus*: phenylethyl alcohol (A5, “fruity,” “rose,” and “sweet”), 2-thiophenemethanethiol (A6, “roasted” and “coffee”), ethanone, 1-(2-aminophenyl)- (A9, “grape” and “sweet”), 2,4-decadienal, (E,E)- (A2, “dusty,” “waxy,” and “oily”), 4-nonenal, (E)- (A13, “fruity”), 2,4-decadienal, (E,Z)- (A14, “fried,” “fatty,” and “green”), indole, 3-methyl- (A3, “fecal,” “indole,” and “civet”), pyridine, 2-pentyl- (A7, “fatty,” “green,” “mushroom” and “herbal”), and 2-methoxy-4-vinylphenol (A11, “spicy” and “raisin”). In contrast, 2,4-undecadienal (A15, “buttery,” “spicy,” “baked,” and “fatty”) decreased during the GR-0d to GR-10d period and was negatively associated with *Lactiplantibacillus*.

*Aspergillus* abundance was positively correlated with the same key aroma compounds as *Lactiplantibacillus*. This significant positive correlation suggested potential synergistic interactions between these two taxa in shaping the aroma profile. A possible explanation is that *Aspergillus*, known for its diverse enzymatic repertoire, may contribute to the breakdown of complex substrates in the pomelo must, potentially releasing precursors that *Lactiplantibacillus* can subsequently convert into aromatic compounds, such as A5 and others (Xu et al., 2024). The concurrent decline of both *Lactiplantibacillus* and the associated aroma compounds after GR-10d further supported a functional link between this microbial consortium and aroma formation, which may be disrupted by environmental stresses, such as acid accumulation and ethanol toxicity in the later stages. Previous studies have indicated that *Aspergillus* is a pivotal constituent of the microbiota contributing to taste characteristics in flat peach wine fermentation (Xu et al., 2024), while *Lactiplantibacillus* predominantly contributes to the production of flavor components in black raspberry wine (Wang et al., 2023). The co-occurrence and correlated metabolic output observed in the present study highlighted a potentially important, yet complex, microbial interaction worthy of future investigation.

*Penicillium* and *Zasmidium* showed significant negative correlations with numerous key aroma compounds. While the underlying mechanism remains to be fully elucidated, this inverse relationship may indicate competition for nutrients or ecological niches with the aroma-producing microbial community, rather than direct inhibition of the aroma synthesis pathways (Zhang et al., 2023). This competitive dynamic may indirectly limit the resources available for the biosynthesis of these desirable aroma compounds by other microbes.

## 4 Conclusion

In the present study, the dynamic changes in physicochemical parameters, volatile metabolite profiles, and microbial community succession were monitored during the semi-inoculated fermentation of pomelo wine. Lactic acid bacteria (*Weissella*, *Pediococcus*, and *Lactiplantibacillus*), fermentation yeasts (*Saccharomyces*, *Komagataella*, and *Wickerhamomyces*), and *Aspergillus* were identified as keystone taxa. An inter-kingdom correlation network further identified tightly linked core microbial consortia, while aroma profiling revealed 17 key differential volatile compounds. Moreover, microbe–volatile metabolite association analyses unveiled a correlative landscape that suggested a model whereby niche competition between fungi and bacteria may be a driving force in shaping the aroma signature of Jinggang honey pomelo wine. This model, which links microbial ecological dynamics to metabolic output, provides a mechanistic hypothesis and a foundation for the future precision modulation of fruit wine flavor.

## Data Availability

The original contributions presented in the study are publicly available. This data can be found here: (http://www.ncbi.nlm.nih.gov/bioproject/1375658/PRJNA1375658).

## Footnotes

1
https://chemdata.nist.gov

## Supplementary material

SUPPLEMENTARY FIGURE S1 Fermentation kinetics curve of pomelo wine. Changes in total soluble solid (TSS) and alcohol contents during fermentation. The data points represent the mean ± standard error (*n* = 3).
