# Supplementary figures and images for: Microbiome–volatile metabolome analysis reveals aroma regulation driven by microbial niche competition in Jinggang honey pomelo wine
Source: Front Microbiol. 2026 Jan 2;16:1725554. doi: 10.3389/fmicb.2025.1725554 (PMC12807929; doi:10.3389/fmicb.2025.1725554)

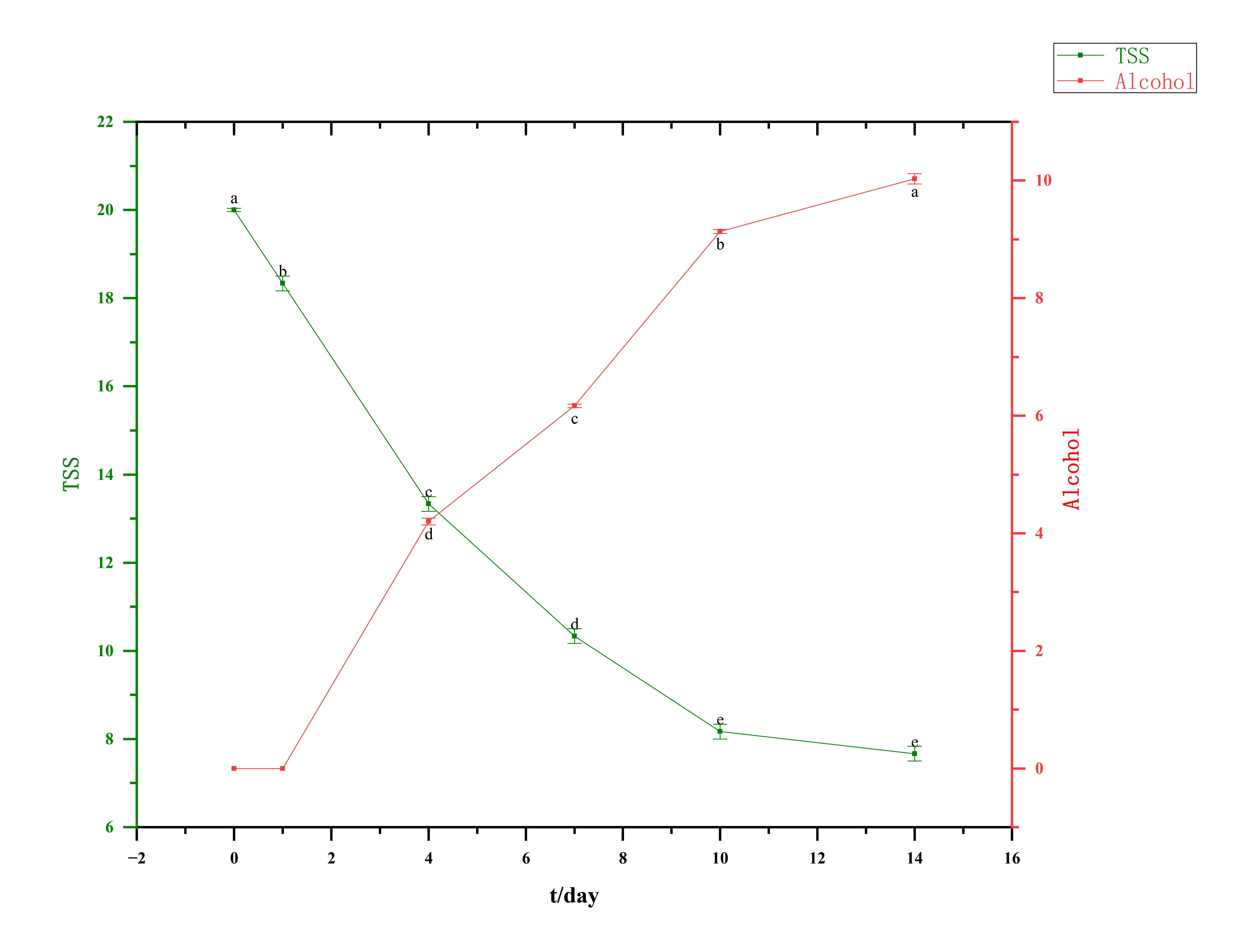

Supplement: SUPPLEMENTARY FIGURE S1 — Fermentation kinetics curve of pomelo wine. Changes in total soluble solid (TSS) and alcohol contents during fermentation. The data points represent the mean ± standard error (n = 3). [file Image_1.JPEG]
